# MFS Transporters and GABA Metabolism Are Involved in the Self-Defense Against DON in *Fusarium graminearum*

**DOI:** 10.3389/fpls.2018.00438

**Published:** 2018-04-13

**Authors:** Qinhu Wang, Daipeng Chen, Mengchun Wu, Jindong Zhu, Cong Jiang, Jin-Rong Xu, Huiquan Liu

**Affiliations:** ^1^State Key Laboratory of Crop Stress Biology for Arid Areas, College of Plant Protection, Northwest A&F University, Yangling, China; ^2^Department of Botany and Plant Pathology, Purdue University, West Lafayette, IN, United States; ^3^Innovation Experimental College, Northwest A&F University, Yangling, China

**Keywords:** Fusarium head blight, *Fusarium graminearum*, DON resistance, MFS transporter, gamma-aminobutyric acid

## Abstract

Trichothecene mycotoxins, such as deoxynivalenol (DON) produced by the fungal pathogen, *Fusarium graminearum*, are not only important for plant infection but are also harmful to human and animal health. Trichothecene targets the ribosomal protein Rpl3 that is conserved in eukaryotes. Hence, a self-defense mechanism must exist in DON-producing fungi. It is reported that *TRI* (trichothecene biosynthesis) *101* and *TRI12* are two genes responsible for self-defense against trichothecene toxins in *Fusarium*. In this study, however, we found that simultaneous disruption of *TRI101* and *TRI12* has no obvious influence on DON resistance upon exogenous DON treatment in *F. graminearum*, suggesting that other mechanisms may be involved in self-defense. By using RNA-seq, we identified 253 genes specifically induced in DON-treated cultures compared with samples from cultures treated or untreated with cycloheximide, a commonly used inhibitor of eukaryotic protein synthesis. We found that transporter genes are significantly enriched in this group of DON-induced genes. Of those genes, 15 encode major facilitator superfamily transporters likely involved in mycotoxin efflux. Significantly, we found that genes involved in the metabolism of gamma-aminobutyric acid (GABA), a known inducer of DON production in *F. graminearum*, are significantly enriched among the DON-induced genes. The GABA biosynthesis gene *PROLINE UTILIZATION 2-2* (*PUT2-2*) is downregulated, while GABA degradation genes are upregulated at least twofold upon treatment with DON, resulting in decreased levels of GABA. Taken together, our results suggest that transporters influencing DON efflux are important for self-defense and that GABA mediates the balance of DON production and self-defense in *F. graminearum*.

## Introduction

Fusarium head blight caused by the fungal pathogen, *Fusarium graminearum*, is a devastating disease of wheat, maize, barley, and other grain cereals worldwide (Bai and Shaner, 2004; Goswami and Kistler, 2004). *F. graminearum* infects the spikelet of cereals and produces harmful mycotoxins during the infection. Thus, it not only causes severe yield losses but also contaminates the infected grains (Tanaka et al., 1988; Placinta et al., 1999). Deoxynivalenol (DON, formerly known as vomitoxin), a trichothecene mycotoxin, is the major secondary metabolite produced by *F. graminearum* and commonly detected in the cereal-based foods (Lombaert et al., 2003; Rasmussen et al., 2003; Tutelyan, 2004; Isebaert et al., 2005). DON severely impacts a number of critical cellular processes in both animals and plants, such as inhibition of protein synthesis, alteration of membrane structure, and inhibition of mitochondrial function (Rocha et al., 2005). It impairs the growth, immunity, and reproduction of human and animals (Pestka and Smolinski, 2005; Bimczok et al., 2007; Pestka, 2010; Sobrova et al., 2010). Besides, DON biosynthesis is important for the infection of host plants (Proctor et al., 1995, 1997), and the aggressiveness of *F. graminearum* is positively correlated with the capacity of DON production (Mesterházy, 2002).

*TRI5*, which encodes an enzyme that catalyzes the first step in the trichothecene biosynthetic pathway, was the first gene identified for trichothecene biosynthesis (Proctor et al., 1997; Brown et al., 2001). *TRI5* is located in the core *TRI* gene cluster on chromosome 2, which represents the major cluster responsible for trichothecene biosynthesis. In the core *TRI* gene cluster, seven genes (*TRI3*, *TRI4*, *TRI5*, *TRI7*, *TRI8*, *TRI11*, and *TRI13*) encode the enzymes required for trichothecene biosynthesis. Two are regulator genes (*TRI6* and *TRI10*), and one (*TRI12*) encodes a major facilitator superfamily (MFS) transporter (Brown et al., 2001; Alexander et al., 2009). Beyond the core *TRI* gene cluster, a mini-cluster formed by *TRI1* and *TRI16* on chromosome 1 and a single-gene of *TRI101* on chromosome 4 are also required for trichothecene biosynthesis.

Trichothecene primarily targets ribosomal protein L3 (Rpl3), which is conserved in eukaryotes, and inhibits protein synthesis by interfering with elongation termination (Wei et al., 1974; Rocha et al., 2005). Thus, trichothecene is not only toxic to animals and plants but also harmful to the pathogen itself. A self-defense mechanism must exist in fungi. *TRI101* and *TRI12* have been shown to provide some degree of resistance to trichothecene (Kimura et al., 1998a,b; Alexander et al., 1999; McCormick et al., 1999). Tri101 acetylates trichothecene to a hypotoxic form by catalyzing the transfer of an *O*-acetyl group at the C3 position (Garvey et al., 2008). Yeast expressing *FgTRI101* is resistant to the trichothecene T-2 toxin (Kimura et al., 1998a), and the expression of *FgTRI101* is induced in T-2 toxin-treated mycelia of *F. graminearum* (Kimura et al., 1998b). Yeast expressing the *TRI101* from *Fusarium sporotrichioides* has also increased tolerance to the trichothecene diacetoxyscirpenol (DAS) toxin (McCormick et al., 1999). Tri12 is an MFS transporter involved in DON efflux (Menke et al., 2012). In *F. sporotrichioides*, the *TRI12* disruption mutant exhibits increased sensitivity to DAS (Alexander et al., 1999). In *F. graminearum*, a *TRI12* disruption mutant was also shown to be sensitive to endogenous mycotoxin in a trichothecene biosynthesis induction (TBI) medium (Menke et al., 2012). Furthermore, transgenic plants carrying *TRI101* have enhanced tolerance to exogenously added trichothecenes (Alexander, 2008).

Although *TRI101* and *TRI12* are involved in the trichothecene tolerance, they are not essential for self-defense. In *F. sporotrichioides*, the spore germination of the *FsTRI12* disruption mutant is unaffected by exogenous DAS (Alexander et al., 1999), and the *Fstri101* deletion mutant is only slightly reduced in radial growth at a high concentration of DAS or T-2 toxin (McCormick et al., 1999). In *F. graminearum*, the radial growth of *FgTRI12* disruption mutant is only slightly reduced compared to the wild-type in TBI medium (Menke et al., 2012). Therefore, beyond *TRI101* and *TRI12*, additional self-defense mechanism may exist in these fungi.

To facilitate the study of the self-defense mechanism in *F. graminearum*, in this study, we constructed a mutant simultaneously disrupted in *TRI5*, *TRI12*, and *TRI101*, and performed RNA-seq analysis of the mutant treated with exogenous DON. The objectives of our study were to determine: (i) whether simultaneous disruption of *TRI12* and *TRI101* results in destruction of DON resistance and (ii) whether additional genes or pathways are involved in the self-defense. We found that knocking out *TRI12* and *TRI101* has no obvious influence on the radial growth and conidial germination with or without exogenous DON treatment. By RNA-seq analysis, we found that transporters are important for DON resistance. Importantly, we found that gamma-aminobutyric acid (GABA) is involved in the self-defense and likely balances DON production and self-defense in *F. graminearum*.

## Results

### Knockout of *TRI5–TRI12* and *TRI101* Blocks DON Production and Plant Infection

Because *TRI5* and *TRI12* are separated by *TRI10*–*TRI9*–*TRI11* in the core *TRI* gene cluster, the entire *TRI5*–*TRI12* (*TRI5*–*TRI10*–*TRI9*–*TRI11*–*TR12*) cluster was replaced with the hygromycin phosphotransferase (*HPT*) gene (Supplementary Figure S1A) in *F. graminearum* strain PH-1, resulting in the *tri5*–*tri12* mutant (strain T512). The *TRI101* gene was subsequently replaced with the neomycin phosphotransferase II (*NPT*) gene in strain T512, and the resulting mutant is designated *tri5*–*12*–*tri101* mutant (strain T512101) in this study (Supplementary Figure S1A). The disruptions of these regions were confirmed by Southern blot (Supplementary Figure S1B). In all of the two replacements, we could detect the inserted selection makers, but none of the targeted DNA fragments were detected (Supplementary Figure S1B). These data clearly confirmed that both targeted regions were successfully replaced.

**FIGURE 1:**
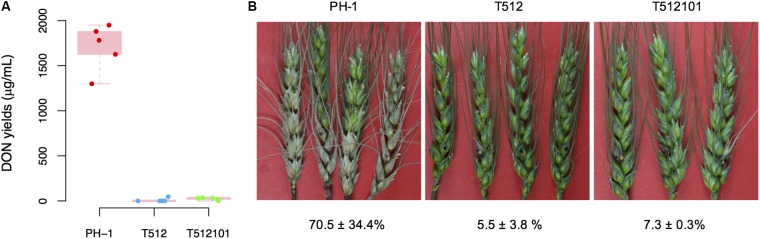
DON production and pathogenicity of the *tri5*–*12* and *tri5*–*12*–*tri101* mutants. **(A)** Boxplot of DON yields assayed on liquid TBI culture for *F. graminearum* strains PH-1, T512, and T512101. For each strain, five replicates were used. **(B)** Flowering wheat heads were drop-inoculated with conidia from the same set of strains. Spikelets with typical symptoms were photographed 14 days post-inoculation. The percentages of infected spikelets are indicated (mean ± SD).

The production of DON in *tri5*–*12* and *tri5*–*12*–*tri101* mutants on liquid TBI medium was measured. As expected, DON production in these two mutants was absolutely abolished (**Figure 1A**), which is consistent with the fact that *TRI5* is essential for DON production in *F. graminearum*. To examine whether the disruption of *TRI5–TRI12* and *TRI101* affects infection of the host plant, we inoculated wheat spikelets with conidia produced by the mutants. Compared to the wild-type of *F. graminearum* strain PH-1, the pathogenicity of the mutants *tri5*–*12* and *tri5*–*12*–*tri101* was nearly lost (**Figure 1B**). By 2 weeks after inoculation, the disease lesions were restricted to the inoculation site and did not spread to the nearby spikelet (**Figure 1B**). These data confirmed that *TRI5*–*TRI12* and *TRI101* are critical for the pathogenicity of *F. graminearum* on wheat heads.

### The *tri5*–*12*–*tri101* Mutant Is Not Hypersensitive to DON Treatment

**FIGURE 2:**
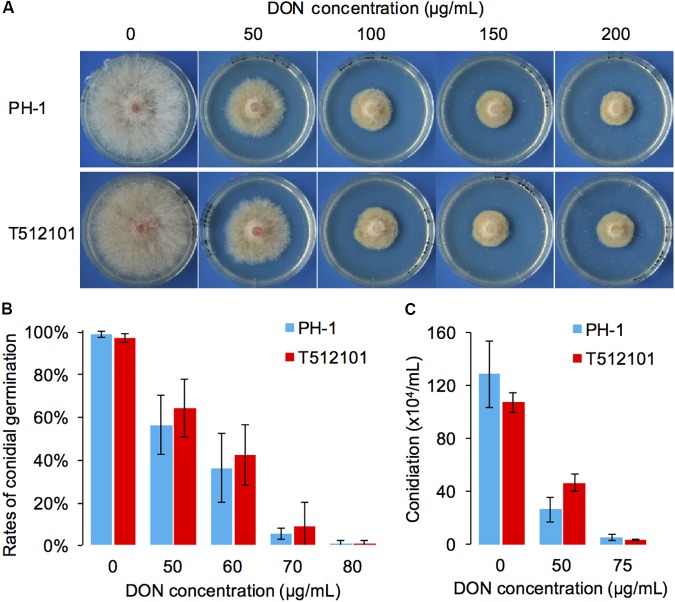
DON resistance assays of the *tri5*–*12*–*tri101* mutant. **(A)** The radial growth of PH-1 and T512101 cultured on YPG medium containing 0, 50, 100, 150, and 200 μg/mL DON, respectively, at 25°C for 72 h. **(B)** Conidial germination of PH-1 and T512101 on YPD medium containing 0, 50, 60, 70, and 80 μg/mL DON, respectively. Error bars represent the SD of three replicates. **(C)** Conidiation of PH-1 and T512101 on CMC medium containing 0, 50, and 75 μg/mL DON, respectively. Error bars represent the SD of six replicates.

Compared to the wild-type PH-1, the two mutants, *tri5*–*12* and *tri5*–*12*–*tri101*, did not show any significant changes in radial growth (Supplementary Figure S2). To examine whether *tri5*–*12*–*tri101* mutant has decreased resistance to DON, the radial growth of the mutant was analyzed in the presence of exogenous DON. Although adding increasing concentrations of DON inhibited the radial growth of the mutant, there was no obvious difference (IC50_PH-1_ = 45.1, IC50*_tri5_*_-_*_12_*_-_*_tri101_* = 52.4) observed between the mutant and wild-type strain (**Figure 2A**). Consistently, the conidial germination of the mutant was inhibited at rates similar to those in the wild-type strain (IC50_PH-1_ = 56.7, IC50*_tri5_*_-_*_12_*_-_*_tri101_* = 59.7) treated with different concentrations of exogenous DON (**Figure 2B**). Similar levels of conidiation inhibition were also observed between the DON-treated mutant and the wild-type (**Figure 2C**). These results suggest that the deletion of both *TRI12* and *TRI101* has no obvious effects on DON resistance in *F. graminearum*.

### RNA-Seq Identification of Genes Specifically Induced by DON Treatment

**FIGURE 3:**
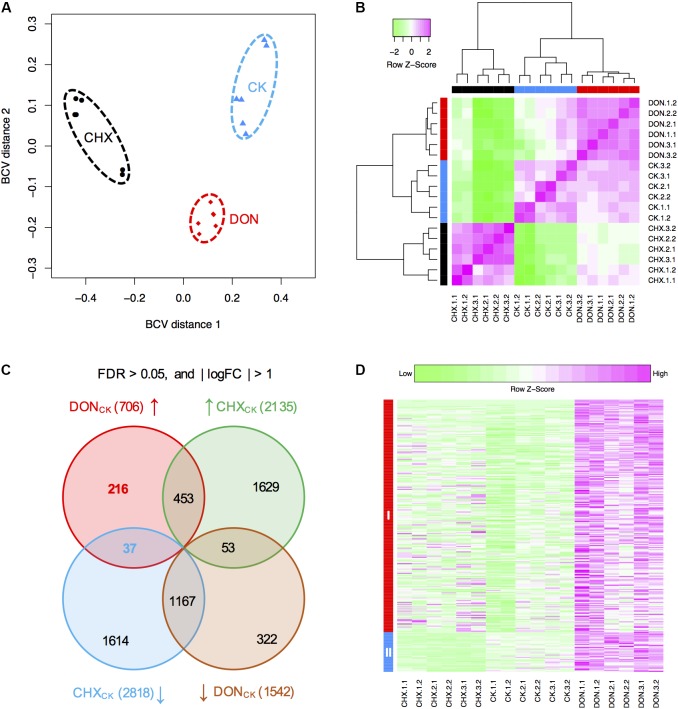
RNA-seq analysis of the *tri5*–*12*–*tri101* mutant. **(A)** Multidimensional scaling plot of the transcriptome profiles of the *tri5*–*12*–*tri101* mutant treated with DON, CHX, or untreated (CK). **(B)** Hierarchical clustering analysis showing the relationship of the gene expression among different samples according to RNA-seq analysis. In **(A)** and **(B)**, the untreated *tri5*–*12*–*tri101* mutant, the mutant treated with DON, or CHX are marked in blue, red, or black, respectively. Each sample contains six replicates, including three biological replicates, and each biological replicate has two technical replicates. **(C)** Comparison of the differentially expressed genes in *tri5*–*12*–*tri101* mutant treated with DON or CHX. The genes upregulated in DON (in red) and CHX (in green) treatments, and the genes downregulated in DON (in blue) and CHX (in orange) treatments are illustrated in the Venn diagram. Up arrows indicate upregulation, while the down arrows indicate downregulation. **(D)** Heatmap showing the expression patterns of the 253 specifically induced genes in the *tri5*–*12*–*tri101* mutant upon DON treatment. Genes in class I (in red) are upregulated upon DON treatment. Genes in class II (in blue) are upregulated upon DON treatment, but downregulated upon CHX treatment.

To identify novel genes conferring self-defense, we next performed RNA-seq to analyze gene expression in the DON-treated or untreated (CK) *tri5*–*12*–*tri101* mutant. Because DON inhibits protein synthesis, the expression of numerous genes not related to DON resistance may be affected. To identify the genes specifically induced by DON treatment, we used cycloheximide (CHX), a eukaryote protein synthesis inhibitor, as a control to filter out the differentially expressed genes from those caused by general inhibition of protein synthesis. For each treatment, three biological replicates were used, and each of the biological replicates contained two technical repeats. We calculated the distances of these 18 samples based on the biological coefficient of variation. As expected, all the samples from the same treatment could be grouped together (**Figure 3A**). Further clustering analysis based on the correlation relationships of these samples also suggested that the genes expressed in different treatments are distinct (**Figure 3B**). Therefore, DON treatment considerably affected the profile of *tri5*–*12*–*tri101* gene expression.

Compared to the CK, 706 and 1,542 genes were upregulated and downregulated, respectively, at least twofold upon DON treatment of the *tri5*–*12*–*tri101* mutant, while 2,135 and 2,818 genes were upregulated and downregulated upon CHX treatment (**Figure 3C**). In total, 253 upregulated (Supplementary Table S1) and 375 downregulated genes were specifically affected by DON treatment (**Figure 3C**). Notably, 37 of these 253 DON-specific induced genes were downregulated even in the CHX samples (**Figures 3C,D**). These specifically induced genes likely confer tolerance and self-defense against DON.

### DON-Specific Induced Genes Are Enriched for Transporter Genes

**FIGURE 4:**
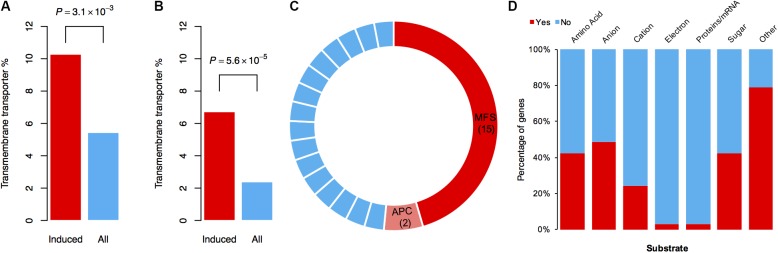
Transporter genes are enriched in DON-specific induced genes. **(A)** The percentages of genes involved in “transmembrane transport” processes or have “transmembrane transporter activity” in the 253 DON-specific induced genes (Induced) or in the total genes (All) according to GO annotation. **(B)** The percentages of genes annotated as a transporter in the 253 DON-specific induced genes (Induced) or in the total genes (All) according to BLAST annotation. In **(A)** and **(B)**, *P*-values were obtained from chi-square tests. **(C)** Classification of 33 specifically induced transporter genes. Fifteen MFS transporter genes and two APC transporter genes are highlighted in red and pink, respectively. Each blue stripe in the circle represents one transporter gene of other families as listed in Supplementary Table S2. **(D)** Percentage of the transporter genes with designated substrates.

To identify which types of genes mainly contribute to the tolerance against DON in the mutant, we performed functional annotation on the specifically induced genes resulting from DON treatment. Interestingly, 17 and 26 genes encoding different types of transporters were found by BLAST and gene ontology (GO) analyses, respectively (Supplementary Table S1). Further statistical analysis revealed that the transporter genes were significantly enriched among these specifically induced genes (**Figures 4A,B**). To obtain a comprehensive understanding of the induced transporter genes, we performed formal transporter annotation. In total, we found that 13% (33 of the 253) of the specifically induced genes were transporter genes (Supplementary Table S2), which is 2.5-fold higher than the ratio of the transporter genes in the genome of *F. graminearum*.

### The MFS Transporters May Be Important for Self-Defense

Notably, among these 33 transporters, 15 of them were MFS transporters, two of them were amino acid–polyamine–organocation (APC) family transporters, and the rest 16 belonged to different transporter families (**Figure 4C**). Tri12 is a known MFS family transporter that exports trichothecene in *Fusarium* (Alexander et al., 1999; Menke et al., 2012). Therefore, it is quite likely that these transporters, or at least some of the MFS family transporters, have a role in pumping out DON and thus reducing the intracellular DON level. Consistently, the homolog of FGRRES_01997 (Supplementary Table S2), which encodes an MFS transporter MgMfs1, is shown to have a role in resistance to toxic compounds and fungicides in *Zymoseptoria tritici* (Roohparvar et al., 2007). A homolog of FGRRES_03033 (Supplementary Table S2), which encodes an MFS transporter Ctb4 in *Cercospora nicotianae*, is required for export of the toxin, cercosporin, and fungal virulence (Choquer et al., 2007). Both these two MFS transporters (FGRRES_01997 and FGRRES_03033) were strongly induced upon various concentrations of DON treatments in *F. graminearum*, as revealed by quantitative real time polymerase chain reaction (qRT-PCR) assays (Supplementary Figure S3). In addition, we found that most of the substrate profiles of these transporters were similar to that of the Tri12 transporter (**Figure 4D** and Supplementary Table S2). Taken together, we conclude that the MFS transporters are important for self-defense against DON in *F. graminearum*.

### DON-Specific Induced Genes Are Enriched in Genes Involved in GABA Metabolism

To identify which processes mainly contribute to the self-defense in the mutant, we performed GO enrichment analysis of the 253 DON-specific induced genes. Interestingly, only two enriched GO terms were found (**Table 1**). The most significant GO term was GABA metabolic process, and the other significant GO term was reactive nitrogen species (RNS) metabolic process. Close examination showed that five (**Table 2**) of the seven genes involved in GABA metabolism and three of the six genes involved in RNS metabolism were specifically upregulated upon DON treatment (**Table 1**). These results suggest that the GABA and RNS metabolic pathways or related genes may contribute to the tolerance and self-defense against DON mycotoxin.

**Table 1:** Enriched GO terms of the 253 DON-specific induced genes.

GO ID	Total	Test	Adjusted *P*	GO term
GO:0009448	7	5	6.9 × 10^-3^	Gamma-aminobutyric acid metabolic process
GO:2001057	6	3	3.2 × 10^-2^	Reactive nitrogen species metabolic process

**Table 2:** DON-specific induced genes involved in GABA metabolism.

Gene ID	Gene name	Function
FGRRES_05554	*GTA-1*	GABA transaminase
FGRRES_06751	*GTA-2*	GABA transaminase
FGRRES_06752	*SSADH-1*	Succinate-semialdehyde dehydrogenase
FGRRES_11843	*SSADH-2*	Succinate-semialdehyde dehydrogenase
FGRRES_04196	*SSADH-3*	Succinate-semialdehyde dehydrogenase

### Reduction of GABA Accumulation as a Mechanism for Self-Defense

**FIGURE 5:**
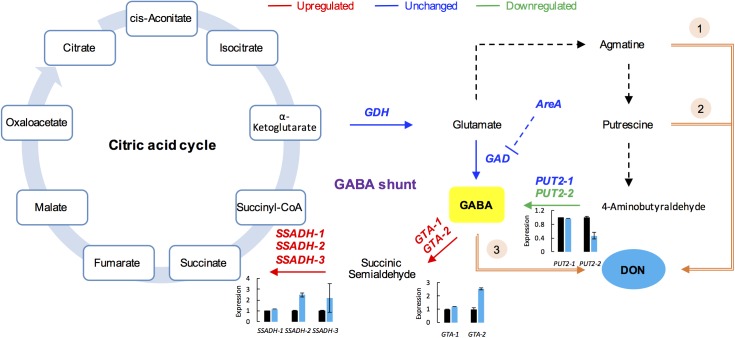
GABA consumption confers self-defense in *F. graminearum*. GABA shunt (middle) bypasses the citric acid cycle (left). In the GABA shunt, glutamate is metabolized to GABA. Various nitrogen substrates, such as agmatine, putrescine, and 4-aminobutyralehyde result in GABA accumulation (right). Furthermore, agmatine (1), putrescine (2), and GABA (3) induce DON production (in orange). In the *tri5*–*12*–*tri101* mutant, the GABA biosynthesis gene *PUT2-2* is downregulated, while the GABA degradation genes, *GTAs* and *SSADHs*, are upregulated upon DON treatment, which results in a decrease in GABA. The gene name in red, blue, and green indicates the gene is upregulated, unchanged, and downregulated, respectively, according the RNA-seq data. Barplots represent the relative expression levels obtained by qRT-PCR in DON treated (blue) and untreated (black) mycelia.

Gamma-aminobutyric acid is a major inhibitory neurotransmitter in animals and also an important signaling metabolite produced by plants and fungi (Kumar and Punekar, 1997; Gilliham and Tyerman, 2016). The GABA pathway is present in fungi (Kumar and Punekar, 1997), and GABA is associated with the DON production in *F. graminearum* (Bonnighausen et al., 2015). The GABA shunt bypasses the citric acid cycle from alpha-ketoglutaric acid to succinic acid via glutamate, GABA, and succinic semialdehyde (SSA; **Figure 5**). In addition, GABA synthesis is induced by agmatine (Lowe et al., 2010; Suzuki et al., 2013), putrescine, and 4-aminobutyraldehyde (Shelp et al., 2012). Further, agmatine (Suzuki et al., 2013), putrescine (Gardiner et al., 2010), and GABA (Bonnighausen et al., 2015) are all positively associated with DON production in *F. graminearum* (**Figure 5**).

To investigate how GABA may contribute to the self-defense, we examined the expression of the GABA metabolic genes in the *tri5*–*12*–*tri101* mutant upon DON treatment according to our RNA-seq data. Interestingly, we found that the two *GTA* genes, which encode GABA transaminases that catalyze GABA to SSA, were upregulated (**Figure 5**). In addition, expression of all of the three *SSADH* genes, which encode SSA dehydrogenase that further catalyze SSA to succinic acid, was also upregulated (**Figure 5**). However, the expression of two genes, *GLUTAMATE DECARBOXYLASE* (*GAD*) and *PROLINE UTILIZATION 2-1* (*PUT2-1*), which contribute to GABA accumulation, was unchanged (**Figure 5**). *PROLINE UTILIZATION 2-2* (*PUT2-2*) was even downregulated in the mutant upon DON treatment (**Figure 5**). The expression of *PUT2*, *GTA*, and *SSADH* genes was further confirmed by qRT-PCR analysis (barplots in **Figure 5**); all of them are consistent with the RNA-seq result.

**FIGURE 6:**
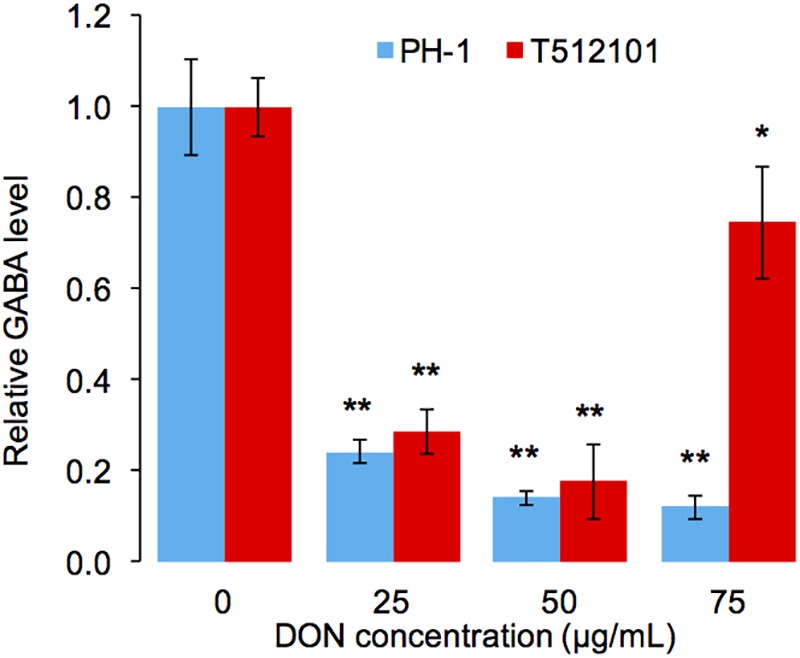
Relative accumulation level of GABA in *F. graminearum*. The level of GABA was normalized to the untreated mycelia (0 μg/mL). Error bars represent the SD of three technical replicates. One-sided *t*-tests were used to access the significances between DON treated and untreated samples, “^∗^” stands for *P*-value < 0.05, and “^∗∗^” stands for *P*-value < 0.01.

To confirm the reduction of the GABA accumulation upon DON treatment, we performed high-performance liquid chromatography (HPLC) analysis in the *tri5*–*12*–*tri101* mutant. Compared to the untreated mycelia, the level of GABA accumulation in the mutant was significantly reduced (**Figure 6**). Similar results were obtained by using the wild-type strain PH-1 (**Figure 6**). Taken together, it is most likely that *F. graminearum* prevents DON accumulation by reducing GABA accumulation.

## Discussion

**FIGURE 7:**
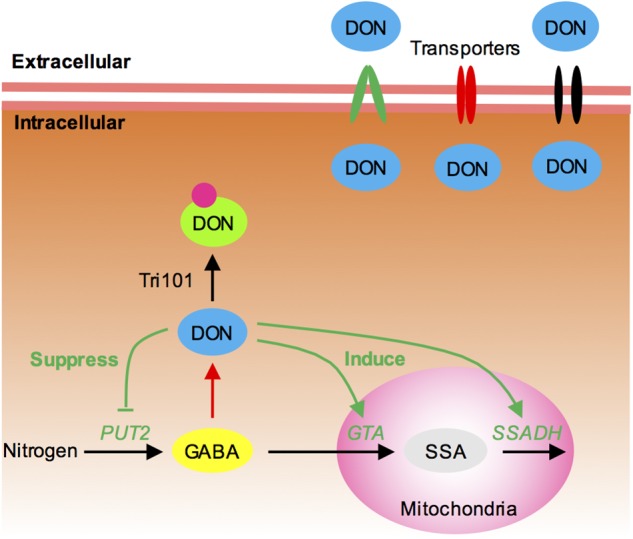
Proposed model for the self-defense mechanism in *F. graminearum*. (1) Tri101, which acetylates DON into a hypotoxic form, is shown to contribute to self-defense. (2) Many MFS transporters, including Tri12, are likely to export intracellular DON. (3) GABA mediates DON production and self-defense in *F. graminearum*. In most cases, increasing GABA accumulation will result in increasing DON production (red line). However, higher concentrations of intracellular DON may feedback inhibit DON production by reducing GABA accumulation (green line). It suppresses the expression of GABA biosynthesis gene *PUT2-2* and induces the expression of GABA degradation genes *GTA* and *SSADH*, preventing further DON accumulation.

Trichothecene is not only toxic to animals and plants but also harmful to the pathogen itself. Therefore, DON-producing fungi must possess a protective self-defense mechanism. *TRI101* and *TRI12* are two self-defense genes for mycotoxin reported in *Fusarium* (**Figure 7**): Tri101 acetylates trichothecene to a hypotoxic form (Kimura et al., 1998a; Garvey et al., 2008), and Tri12 pumps trichothecene to the extracellular zone of the cells (Alexander et al., 1999; Menke et al., 2012). In this study, we investigated whether simultaneous disruption of *TRI12* and *TRI101* could result in severe impairment of DON resistance in *F. graminearum*. Unexpectedly, our results suggested that the *tri5*–*12*–*tri101* mutant was not hypersensitive to exogenous DON treatment compared to wild-type PH-1. In fact, previous studies showed that *TRI12* or *TRI101* knockout mutants are not significantly impaired in toxin resistance (Alexander et al., 1999; McCormick et al., 1999; Menke et al., 2012). In addition, previous self-defense assays for *TRI101* were conducted in *F. sporotrichioides* or yeast, and used T-2 or DAS, but not DON toxin treatment (Kimura et al., 1998a; McCormick et al., 1999). The self-defense role for *TRI12* in *F. graminearum* was analyzed on TBI medium, rather than by treatment with exogenous DON (Menke et al., 2012). It is possible that one or two other genes may play a minor role in the self-protection against mycotoxin in *F. graminearum*. By using the *tri5*–*12*–*tri101* mutant, we identified 253 genes specifically induced upon DON treatment relative to CHX treatment. The transporter genes and GABA metabolism genes are enriched among these DON-specific induced genes, suggesting that they are most likely involved in the establishment of DON resistance for *F. graminearum*.

Transporters provide a channel for drug and mycotoxin efflux. Indeed, Tri12 is an MFS transporter (Menke et al., 2012). Many MFS transporters and ATP-binding cassette (ABC) transporters have been shown to contribute to toxin efflux in fungi (Coleman and Mylonakis, 2009). In this study, 33 transporters were identified in the DON-specific induced genes. Of those, two were homologous to toxin-efflux-related MFS transporters Cbt4 and MgMfs1, respectively. Many transporter genes were also identified in a trichodiene (the chemical product of Tri5)-treated *TRI5* deletion mutant (Seong et al., 2009). Therefore, transporters, especially MFS transporters, likely cooperate and collectively contribute to DON resistance (**Figure 7**).

Intriguingly, we found that GABA also likely contributes to self-defense in *F. graminearum* (**Figure 7**). It has been shown that GABA positively regulates DON production (Bonnighausen et al., 2015). For example, metabolome analysis of *Fusarium* in both toxin-producing and in non-producing conditions revealed that the concentration of GABA is elevated in the DON-inducing medium (Lowe et al., 2010). Adding of GABA to culture medium strongly induces DON biosynthesis in *F. graminearum* (Bonnighausen et al., 2015). In *Fusarium asiaticum*, addition of GABA inducible agmatine to the culture medium also resulted in significantly high levels of DON production (Suzuki et al., 2013). In this study, however, we found that the *GTA* genes that metabolize GABA are upregulated, and *PUT2-2* that leads to GABA accumulation is downregulated in the DON-treated *tri5*–*12*–*tri101* mutant. Hence, the convergent behaviors of these genes reduce the intracellular level of GABA (**Figure 7**). It seems logical to conclude that DON production and self-defense are balanced by GABA (**Figure 7**). Generally, GABA triggers DON production, but when DON accumulates at a higher level, the fungi may protect themselves by preventing GABA accumulation thereby reducing DON accumulation (**Figure 7**). Besides, the upregulation of the transporter genes and reduction of GABA level upon DON treatment in *F. graminearum* may tightly linked together. In plant, it is shown that GABA negatively regulates the activity of plant-specific anion transporters (Ramesh et al., 2015). It is also possible that, in addition to preventing/switch-off DON accumulation, GABA metabolism dynamics may also be related to the upregulation of the transporter to pump out the mycotoxin when DON accumulates at a higher level.

## Materials and Methods

### Strains and Culture Conditions

*Fusarium graminearum* (*Gibberella zeae*) strain PH-1 and mutant strains T512 and T512101 were cultured on potato dextrose agar (PDA) medium at 25°C. For radial growth determinations, strains were cultured on PDA, complete minimal (CM), or YPG (0.3% yeast extract, 1% peptone, and 2% glucose) medium at 25°C for 72 h. Conidial germination rates were determined on YPD (1% yeast extract, 2% peptone, and 2% dextrose) medium at 25°C for 8 h. Conidiation was assayed on 5-day-old carboxymethylcellulose (CMC) cultures.

### Generation of the *tri5–12–tri101* Deletion Mutant

The deletion mutants were prepared by the split-marker method as described previously (Catlett et al., 2002). Briefly, to generate the *tri5*–*12* mutant, the upstream and downstream flanking sequences were amplified from genomic DNA, and fused with the *HPT* gene by overlapping polymerase chain reaction (PCR). The fusion constructs were transformed into protoplasts of PH-1 by the method described previously (Zhou et al., 2011). The generation of the *tri5*–*12*–*tri101* mutant was similar to procedures used for the *tri5*–*12* mutant, except that the target gene was replaced by the *NPT* gene, and strain T512 was used for transformation. The putative knockout mutants were screened by PCR and further verified by Southern blot hybridizations.

### Plant Infection and DON Production Assays

Conidia harvested from 5-day-old CMC cultures were resuspended in sterile distilled water to a final concentration of 1.0–1.5 × 10^5^ spores/mL as described previously (Jiang et al., 2016). The flowering wheat heads of wheat cultivar Norm were drop-inoculated with 10 μL of conidial suspensions as described previously (Jiang et al., 2016). DON production in liquid TBI cultures was assayed as described previously (Jiang et al., 2016).

### CHX and DON Treatments of the *tri5–12–tri101* Mutant

The conidia of the *tri5*–*12*–*tri101* mutant were harvested from 5-day-old CMC medium and shaken in liquid CM medium at 25°C for 72 h. The germinated conidia were then collected and transferred to the liquid CM medium with 75 μg/mL DON or 10 μg/mL CHX, respectively. After 5-day shaking, the freshly developed mycelia were collected for RNA-seq analysis. Notably, upon treatment with 75 μg/mL DON or 10 μg/mL CHX treatment, the germination of the conidia was nearly 100% inhibited in *F. graminearum* strain PH-1.

### RNA-Seq Analysis

Total RNAs isolated from the DON-treated, CHX-treated, and untreated (CK) mycelia were sequenced with an Illumina HiSeq 2500 at Shanghai Biotechnology Corporation. For each sample, three biological replicates with two technical replicates were sequenced. RNA-seq data were deposited in the NCBI SRA database under accession number SRP120765. The clean data were mapped to the genome of *F. graminearum* (King et al., 2015) by using hisat2 (Kim et al., 2015) with default parameters. The differentially expressed genes (log_2_FC > 1 and FDR < 0.05) were carried out based on the read counts by edgeRun with TMM normalization (Dimont et al., 2015) as described previously (Wang et al., 2017). GO annotation was carried out with Blast2GO (Conesa and Gotz, 2008), and GO enrichment analysis was performed by the parent–child union method with Benjamini–Hochberg correction as developed in Ontologizer (Bauer et al., 2008).

### Transporter Annotation

To identify the transporter genes in DON-specific induced genes, a BLAST search (*E*-value < 1e-10) was first performed against the curated transporter classification database (Saier et al., 2016). To remove the false positive predictions, the candidate transporters and their potential substrates were further examined by the AAindex and PSSM-based methods as developed in TrSSP (Mishra et al., 2014).

### qRT-PCR Analysis

The qRT-PCR was performed as previously described (Wang et al., 2016); 1 μg of total RNA was treated with DNase I and subjected to the first strand cDNA synthesis. Since the expression of the commonly used reference gene *GzUBH* and *BTUB* is changed upon DON treatment, FGRRES_00746 was selected as a reference gene according to the RNA-seq data generated in this study. All the primers used for qRT-PCR analysis are listed in Supplementary Table S3.

### HPLC Analysis of GABA

High-performance liquid chromatography analysis of GABA in mycelia was performed as previously described (Bonnighausen et al., 2015). In brief, GABA was extracted by water/chloroform/methanol (3:5:12, v/v/v) from mycelia, the derivatization was performed with *o*-phthalaldehyde (OPA), and the OPA derivatized samples were detected at 338 nm.

## Author Contributions

HL, J-RX, and QW conceived and designed the experiments. DC, MW, JZ, and QW performed the experiments. QW and HL analyzed the data. QW wrote the manuscript. HL and CJ improved the manuscript.

## Conflict of Interest Statement

The authors declare that the research was conducted in the absence of any commercial or financial relationships that could be construed as a potential conflict of interest.
