# Supplementary material for: MFS Transporters and GABA Metabolism Are Involved in the Self-Defense Against DON in Fusarium graminearum
Source: Front Plant Sci. 2018 Apr 13;9:438. doi: 10.3389/fpls.2018.00438 (PMC5908970; doi:10.3389/fpls.2018.00438)
Supplement: Supplementary file 2 [file Image_1.PDF]

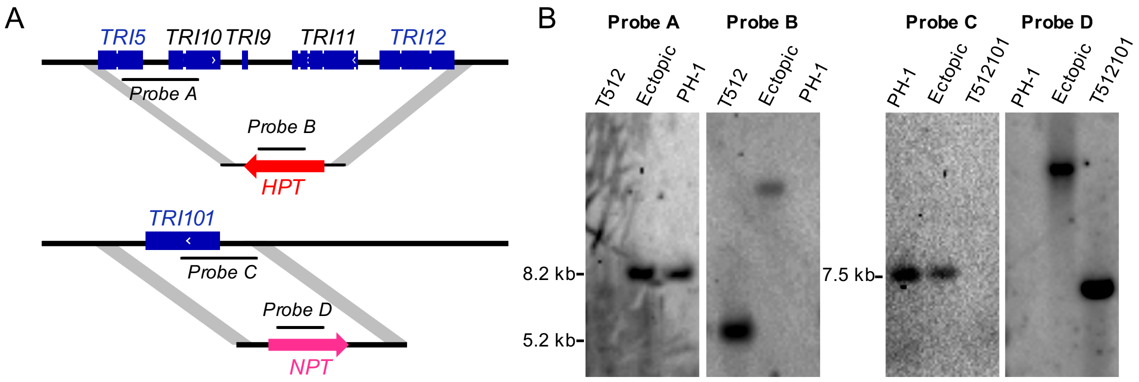

Supplementary Figure 1. The generation of *tri5-12-tri101* mutant. (A) Schematic diagram showing the strategy used for knockout of *TRI* genes. The *TRI5-TRI12* cluster was replaced by the *HPT* gene, and *TRI101* was replaced by the *NPT* gene. (B) Southern blot analysis of the knockout mutant. Probes A and B were used to detect *TRI5-TRI12* and *HPT* genes, respectively, in *F. graminearum* strain PH-1, T512, and the ectopic transformant. Probes C and D were used to detect *TRI101* and *NPT* genes, respectively, in *F. graminearum* strain PH-1, T512101, and the ectopic transformant.

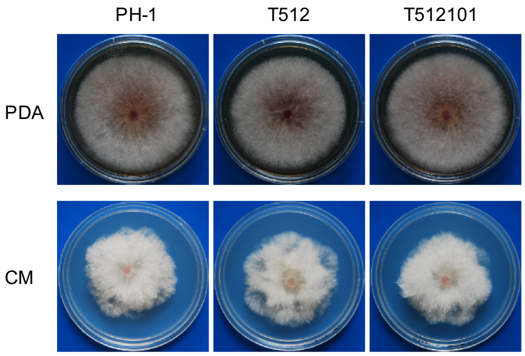

Supplementary Figure 2. The *tri5-12* and *tri5-12-tri101* mutants have radial growth similar to the wild-type strain. These strains were cultured on PDA and CM medium for 72 hours. The wild-type strain PH-1 of *F. graminearum* served as the control.

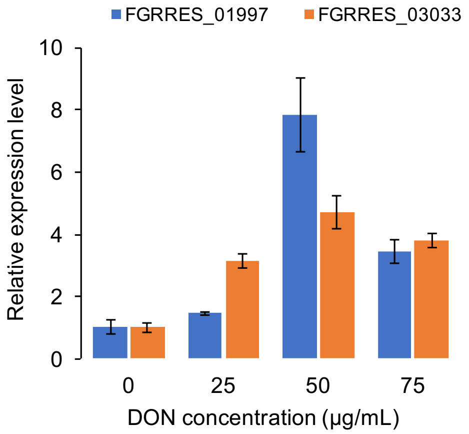

Supplementary Figure 3. The expression of FGRRES_01997 and FGRRES_03033 in DON treated mycelia in strain T512101. The expression levels of these two transporter genes upon DON treatment were normalized to that of the untreated mycelia (0 µg/mL), respectively. Error bars represent the standard deviation of three technical replicates.
